# Brunonianism, Toddism, and Other Isms

**Published:** 1861-01

**Authors:** Samuel D. Gross

**Affiliations:** Professor of Surgery in the Jefferson Medical College


ART. I.—*Brunonianism, Toddism, and other 'Isms.* A paper read before the Philadelphia County Medical Society, November, 14th, 1860. By SAMUEL D. GROSS, M.D., Professor of Surgery in the Jefferson Medical College.

THE revolutions in medicine have been almost as numerous as the revolutions of empires, and hardly, if indeed any, less disastrous in their effects upon human life. The checkered arch which spans its horizon has everywhere inscribed upon it, in legible characters, the words change, change, change. Every age has had its medical absurdities and inconsistencies; and, judging from the tendencies of our own, it is evident that the times are sadly out of joint in these particulars. As fast as one delusion, enslaving the professional mind, disappears, another springs up to take its place; and thus it probably ever will be, for fickleness and instability are written upon all human things. God and truth alone are immutable.

Bacon was a firm believer in charms and amulets; Martin Luther thought there was great efficacy in toads; Boyle held that the thigh-bone of an executed criminal was a specific for dysentery; Berkeley humbugged his countrymen with the virtues of tar-water; and, in comparatively recent times, Perkins set the world agog with his metallic tractors. Mesmerism was rampant in France in the latter part of the last century, and after having been almost forgotten, reappeared in this country, in all its pristine vigor, not more than twenty years ago, under the fostering care of more than one *eminent* professor. Clairvoyance, too, has had its day; while Homœopathy, Chrono-Thermalism, Thompsonianism, Hydropathy, and other kindred delusions are stalking through the land, entrapping the credulous and unwary, and consigning thousands of human beings annually to a premature grave. In Germany and Switzerland the whey and grape cures are at this moment all the rage in the treatment of chronic diseases. What the next humbug will be, time will soon determine.

To a person of a calm, sober, and reflecting mind, all this does not appear strange. Such a result is perfectly natural; a necessary consequence of man's credulity, weakness, and craftiness; of the imperfections of his observing and reasoning faculties. Diversity of thought and action is one of the great prerogatives of our nature. The saddest calamity that could befall the human race would be that in which all men should regard objects from one common stand-point. All efficient progress would instantly cease. The mental world would sink into inertia. Providence has wisely ordained that there should be conflicts of sentiment and varieties of pursuit. It is only when these diversities assume a monstrous form that they are liable to become objects of mischief, involving the best and dearests interests of society. Medical science has largely participated in these blessings and these evils; it has always had its ebbs and flows, its currents and counter-currents. The greatest divergence of opinion still prevails concerning the pathology and treatment even of many of the more common diseases. The profession is full of skeptics. Is it surprising, then, that some master-spirit should occasionally rise up to startle the world, and to dazzle it with the rays of his genius, uprooting, like the storm that sweeps over the earth, and demolishes nature's fairest works, all our preconceived ideas of pathology, and thus unfitting us for the adoption of a rational therapeutics? Such an occurrence cannot, in the very nature of things, be expected to be infrequent; and all experience has shown that the lesser lights of the profession are ever ready to gather round the new luminary as moths gather round the flame of a candle. With little or no thought of consequences, with no pains to investigate the claims of the new candidate to public favor, and dead to the promptings of common sense, they fall an easy prey to every new doctrine, or new mode of practice, however visionary and absurd. Novelty is with them a new species of existence, a kind of hysterical illusion, which they hail as a pleasant change, because it is always followed by excitement, so necessary to their happiness. Their motto, forthwith, is "jacta est alea," and they accordingly enroll themselves under the new banner with all the ardor and enthusiasm of patriots, if not martyrs.

The very instability, then, of medical science and doctrine is often, if indeed not generally, attended with salutary results. The inquiry which is sure to follow in its wake seldom fails to put the erring mind again into its proper track. Like a drunken peasant on horseback, to use one of Luther's similes, it may now be on this side of the saddle, and now on the other, but it will be sure, in the end, to recover its equilibrium. The atmosphere of medicine, like that of law, politics, and even divinity itself, requires occasional ventilation.

If there were no changes in medical doctrine and practice, there certainly could not be any healthful professional progress; the science would become stagnant, and be incessantly in danger of relapsing into barbarism. There is, then, I repeat, something salutary in these revolutions, something positively useful; and yet it cannot, on the other hand, be denied that they often carry with them much that is absolutely hurtful, both as it respects the interest of medical science and the good of the human race.

Considering the frequency of these revolutions, their pervasive influence, and the vastness of their importance not only to the profession, but to mankind at large, it is surprising that there is not in the English language a solitary work in which they are comprehensively and philosophically discussed. Nowhere are they brought out in bold and prominent relief. To the scholar of leisure, and of the requisite clinical experience, there is, in my opinion, no field, the thorough and successful exploration of which holds out so many inducements for exertion; so much prospect of reimbursement in fame and usefulness. To do full justice to the enterprise, the individual should bring to it the possession of vast learning, a discriminating mind, and untiring industry, united with an ample practical acquaintance with medicine and the collateral sciences, and an ardent love of his profession. It should be the work of a long lifetime; anything short of this would only end in disappointment. The study would demand a thorough knowledge of contemporaneous history, an extensive acquaintance with the different sects of philosophy, and a profound inquiry into the character of disease as it has prevailed in different ages, and in different parts of the globe, with an account of the modifying influences of climate, soil, society, and occupation.

It requires no prophetic eye, no special forecast, to discover that we are on the very verge of one of the most fearful and wide-spread revolutions in medicine that the world has ever witnessed; another of those astounding changes whose name is already legion, and which, whether for good or for evil, is destined to convulse the profession, and to exert a most powerful influence upon the practice of the healing art. If this statement be true, as the sequel will show it is, is it not proper that we should pause and inquire, "Watchman, what of the night?" Is it right that we should calmly fold our arms and be silent spectators of the scenes around us, without taking an active interest in them? There is an impending crisis in our profession, and it is the solemn duty of every medical practitioner to meet this crisis in a manly and philosophical spirit. The question is not one of an ephemeral character, or one of unconcern; its solution is of the deepest moment, involving the health, the lives, and the happiness of millions of human beings, born and unborn. Its correct solution is of far greater consequence, in its present and remote bearings on the civilized world, than any political question that has been agitated during the last half century. If this crisis cannot be averted, its crushing effects may be incomparably greater than those of the bloodiest war, or of the most unrelenting epidemic that has for ages spread its desolating mantle over the earth.

The tendency on the part of the medical profession, both in Great Britain and in this country, at the present moment, is the abandonment of all active depletory measures and the employment of stimulating ones, upon the assumption that there has either been a material change in the type of disease, or that there is in all inflammatory affections a peculiar toxic state of the blood, rendering mankind incapable of bearing the same active treatment as formerly. So deeply rooted has this opinion become that many practitioners have already entirely discarded the use of the lancet, purgatives, tartar emetic, and other depressants. Some of them even coincide with the late Dr. Todd, of London, that leeches can no longer be applied with safety.

In order to do anything like justice to this subject, it is necessary to inquire, first, whether there has really been any change in the type of disease, as has been so confidently asserted by some of the advocates of this doctrine; and, secondly, whether there is any foundation for the belief that people are less capable now than formerly of resisting the effects of active depletory remedies. I shall endeavor to show, during the progress of the discussion, that both these views are untrue; or, what is the same thing, that they have had their origin in misconception and superficial observation, if not in a willful disposition to misrepresent and distort facts, in order to render them subservient to the creation of a new theory in medicine, and the establishment of a temporary notoriety.

The idea of the exclusive employment of stimulants in the treatment of inflammatory affections is not new. To go no further back than the last century, it will be found that it enjoyed a remarkable popularity, not only in England, but also in certain parts of the Continent of Europe, especially in Italy and Germany. Under the name of the Brunonian system, it exercised an extensive sway, for nearly twenty-five years, over the minds of many otherwise enlightened practitioners, whose enthusiasm seemed to have been carried away by it to the utmost stretch of the imagination. Many of them, indeed, supposed that they had actually discovered the philosopher's stone, in regard to the treatment of disease, and they openly thanked God that they had been made the humble recipients of this great blessing. John Brown, the author of this practice, was almost deified. He pretended to have made a stupendous discovery, founded mainly upon reflection and his own personal suffering. All diseases were regarded by him as sthenic and asthenic; this was his only classification, and the treatment which he erected upon it was essentially stimulant or supporting.

The biography of this remarkable personage deserves brief notice in connection with his celebrated doctrine, inasmuch as it will serve to bring out the latter in somewhat bolder relief, and show how easy it is for a man of genius to become the founder of a new sect.

John Brown was descended from poor, illiterate parents, his father having been an ordinary day-laborer. He was born at Bunckle, in Berwickshire, Scotland, about 1736. At an early age he was bound as an apprentice to a weaver, but having no taste for the loom he soon revolted and left his master. He was next sent to the grammar school at Dunse, where he pursued his studies with so much ardor and success as to be universally regarded as a prodigy. In the summer he worked in the field in order that he might thus secure the means of improving his mind, which was now the chief object of his ambition. After struggling on in this manner for some years, he became an assistant in the school, and finally resolved to enter the ministry, attaching himself to the sect of Seceders. His habits at this time are described as having been most regular, and so extraordinary was his piety that it was supposed he would become a great and shining light in the church. By degrees, however, he relaxed in religious enthusiasm, and, uniting himself with the Scotch establishment which he had originally so much detested, he soon renounced divinity altogether. He remained at the grammar school till near the age of twenty, earning great reputation as an accomplished scholar, being especially distinguished for his uncommon proficiency in Latin, which he read, spoke, and wrote with great fluency, if not positive elegance. Determined to turn his knowledge to advantage, he settled at Edinburgh as a teacher of Latin, and as a translator of English into Latin theses; in other words, John Brown became what was then and is still known as a *grinder*. He also attended the medical lectures in the University; and in order to support himself and his family—for he was now married—he opened a boarding-house for medical students. In time he became intimate with Cullen, who was so fascinated with his talents and attainments that he was induced to employ him as a private tutor in his family, and at length permitted him to give an occasional evening lecture, in which he had the assistance of the notes of the professor's morning discourse. The friendship, however, which had thus sprung up, was of short duration; Brown's boarding-house scheme failed to afford him the means of a livelihood, and being disappointed in obtaining the chair of Theoretical Medicine in the University, in his application for which he had largely calculated on the influence of Cullen, an open and permanent rupture took place between them, followed by a determined enmity on the part of the protegé toward all the medical professors of Edinburgh.

Thus thrown again upon his own resources, he lost no time in delivering a course of opposition lectures, in which he ridiculed the teachings of Cullen, and gradually developed his new doctrine, finally embodying its principles in his "Elements of Medicine," published in 1780. His time was now chiefly spent in expounding his new theory, and in carousing with his dissolute companions, of whom he had not a few, both in and out of the profession. He was frequently intoxicated, and, neglecting his business, he became so reduced in circumstances as to be committed to prison for debt, where, such were his powers of fascination, his pupils continued to attend his lectures. Disgusted with Edinburgh—for he had now neither friends, nor practice, nor money—he quit Scotland and settled in London, in the hope that a change of residence might bring with it a change of fortune. But, alas! no such result ensued. His career in the English metropolis was as short as it was inglorious. Worn out by intemperance, chagrin, and disappointment, he died, in utter poverty and obscurity, in 1788, at the age of fifty-two, of apoplexy, brought on by an over-dose of laudanum, taken, as some have supposed, for the purpose of self-destruction.

John Brown was a firm and enthusiastic believer in the truth and value of his own doctrine. Having early become a victim to gout, he daily took large quantities of alcohol and laudanum to relieve his suffering, and the consequence was that he was seldom entirely sober. On one occasion, in order to afford a practical illustration of his views, he invited, as he himself declares, some of his most zealous and intelligent disciples to dinner. Before they sat down to the table, he hobbled about the room with great difficulty, his leg being swelled and so excessively painful that he was unable to put it upon the floor; but after the bottle had been freely circulated, and the master had become partially intoxicated, he recovered the use of the parts so completely as to move them with the greatest facility. When engaged in lecturing, he generally had at hand a bottle of whisky and a phial of laudanum, taking a glass of the former and from forty to fifty drops of the latter whenever he felt at all languid, repeating the dose perhaps not less than four or five times during the exercise. "Between the effects of these stimulants," says Dr. Beddoes, one of his biographers and most enthusiastic admirers, "and voluntary exertion, he soon waxed warm, and by degrees his imagination was exalted into frenzy."

Such is a brief sketch of the life and character of John Brown, the founder of the so-called "Brunonian system of medicine." That he was a man of genius and possessed of a vivid imagination, with a superior classical education, his career abundantly attests. That he was lax in his principles and debauched in his habits is equally true. Moreover, he was vain, ambitious, unscrupulous, and not always truthful. One of his greatest sins was ingratitude toward Cullen, who, notwithstanding Brown's assertion to the contrary, was for many years his sincere and devoted friend, and who spared no pains to advance his interests so long as he could without compromising his own dignity. "But," to quote the language of Beddoes, "friendships originating in protection are very prone to terminate in enmity, unless difference in rank and pursuit totally precludes competition; and it is well known that the friendship in question was far from permanent."

The opinions of Brown exerted so wide a sway in Great Britain as to affect, for a time, the whole practice of medicine. In Italy his works were translated by the celebrated Rasori, with notes and annotations; and so great was their influence in that country that there was hardly an intelligent pupil who did not openly avow himself as a Brunonian. The "doctrine" also speedily found its way into Germany and France, in both of which, but especially the former, it made numerous converts, and long maintained its hold upon the profession. In a number of the continental universities its principles were formally expounded by the professors. In Germany, writes an anonymous author, Brown was hailed as the medical Luther, and so great were the dissensions among the pupils of some of the leading schools in regard to the claims of the Northern Light, that it was not uncommon for them to come to blows. At Göttingen the Brunonians were so rebellious as to require the authority of the military to keep them in subjection. Of the extent to which the new doctrine had enslaved some of the German practitioners, a correct idea may be formed when it is stated that, at the hospital at Bamberg, the attending physician, Dr. Marcus, a man of eminence, considered himself justified in distributing, as an average allowance, among nearly five hundred patients affected with various kinds of diseases, a drachm of opium, with three times that quantity of camphor and a pound of alcohol, together with numerous other stimulants.* This was at the close of the last century. In 1782, two years after the publication of the "Elements,"

* Edinburgh Medical and Surgical Journal, September, 1860, p. 259.

Brunonianism had reached its culminating point at Edinburgh. Poland, Russia, and Spain also suffered from the infection. In time it penetrated even into India, its most able and enthusiastic advocates in that country being Dr. William Yates and Dr. Charles Maclean, of the General Hospital at Calcutta. These physicians gave an able and lucid exposition of the new doctrine in a short tract, published in the latter part of the last century, under the title of "A View of the Science of Life on the Principles established in the Elements of Medicine, by the late celebrated John Brown, M.D."

In the United States the works of the founder of the Brunonian system were republished at Portsmouth, New Hampshire, in 1803, and were extensively read by the physicians of that period, not a few of whom became its converts, either wholly or in part.

The fundamental propositions of John Brown are few and simple. "Life," he says, "is a forced state;" and he calls the quality or principle on which its phenomena depend excitability. This excitability, of which a certain amount is allotted to every animated being, is seated in the medullary portion of the nerves and in the muscles, and varies not only in different classes of animals, but in the same animal at different times. When most intense, the animal is most vivacious or susceptible of the action of exciting powers, as the various external impressions—heat, air, food, wine, poisons, contagion, the blood, and secreted fluids—or muscular exertion, the passions and mental emotions. The excitement thus occasioned may be too great, too small, or in just measure. When too great, the excitability becomes defective, and weakness is induced, called indirect debility; when too small, as when the exciting powers or stimulants are withheld, direct debility is the result, because the excitability then exists in excess. Every power that acts on the living frame is a stimulant, and if this exciting power is withdrawn, death ensues as certainly as when the excitability is gone. When the excitement is natural, or in just proportion, health is the result. All diseases, according to this system, are sthenic or asthenic; that is, they are attended either with excitement or with exhaustion.*

The stimulant treatment of John Brown was, as has already been stated, essentially based upon the relief which it afforded him in his arthritic attacks. His first seizure occurred when he was in the thirty-sixth year of his age, in consequence, as he alleges, of abstemious living during the previous six months. Subsequently he noticed that he was peculiarly prone to this kind of suffering whenever he relaxed in his

* Beddoes's edition of Brown's Elements of Medicine, p. lxii. Portsmouth, N. H., 1803.

dietetic habits, until at length he became satisfied that the best remedies for his complaint were nutritious food and alcoholic stimulants. These facts are all clearly stated in the preface to the original work, the Elementa Medicinæ. "The author of this work," says he, "has spent above twenty years in learning, teaching, and diligently scrutinizing every part of medicine. The first five passed away in hearing others, in studying what he had heard, implicitly believing it, and entering upon the possession as a rich and valuable inheritance. His employment, the next five years, was to explain more clearly the several particulars, to refine and give them a nicer polish. During the five following years, nothing having succeeded to his satisfaction, he grew indifferent to the subject; and, with many eminent men, and with the very vulgar, began to deplore the healing art as altogether uncertain and incomprehensible. All this time passed away without the acquisition of any advantage; without that which of all things is the most agreeable to the mind, the light of truth; and so great and precious a portion of the short and perishable life of man was totally lost. He was, at this period, in the situation of a traveler in an unknown country, who, after losing every trace of his way, wanders in the shades of night; nor was it till between the fifteenth and twentieth year of his studies that a faint gleam of light, like the first break of day, dawned upon him."

It is hardly necessary to say that the doctrines of Bennett, Todd, and others, in regard to the treatment of inflammation by stimulants, now attracting so much attention, are essentially the tenets of John Brown, either bodily, or under certain modifications. Mr. Bennett is not, it is true, like Alison, a believer in a change in the type of disease, but his treatment is essentially supporting in almost every form and stage of disease; and a similar practice is inculcated, only more pointedly and with less reservation, by the late Dr. Todd, who, however, avowedly discards the idea of sthenic and asthenic affections insisted upon by Brown. But Brown, although he divided all maladies into these two great classes, was rarely, if ever, known to employ the lancet and other depressants; on the contrary, his practice was essentially stimulant; it was always so in his own person, and in the few cases which were under his charge at Edinburgh; for, be it remembered, John Brown had hardly any professional business, and he was known in the medical circles of the Scottish metropolis merely as a theorist. If I am correct in my conclusions in regard to the Brunonian practice, it follows, as a necessary consequence, that it was essentially similar to the practice so strenuously inculcated at the present day; it certainly amounts to the same thing, only that the modern school carry, if possible, the stimulant treatment still farther than the Brunonians did.

Taking Dr. Todd as a type of the new school, let us briefly inquire into the character of his pathology and therapeutics, as set forth in his work on acute diseases, published only a few days before his lamented death, in 1859.

An attentive perusal of this treatise conclusively shows that he was a thorough and uncompromising humoralist. His pathology was based essentially upon the assumed existence of a poison—*materies morbi*—in the blood, for the elimination of which he employed sustaining measures in all acute inflammations from the very commencement of the attack, hoping thereby to anticipate and prevent depression of the vital powers. Starting with this fundamental doctrine, he contends that all diseases are necessarily asthenic, inasmuch as they always cause undue waste of tissue; that there is a tendency in most, if not in all, diseases to a natural cure; and that the treatment of the physician can be of use only in so far as it may assist in neutralizing the poison upon whose presence and operation in the system diseases depend; in other words, that our drugs or remedies are mere antidotes, incapable of exerting any other influence over morbid action. At the head of the list of stimulants he places alcohol, which, if we are to believe him, not only increases the animal temperature and strengthens the heart, but acts as an aliment, nourishing and sustaining the nervous system in a manner and in a degree superior to those of any other kind of food. One of the most important effects ascribed to it by Dr. Todd, in the treatment of all acute inflammatory diseases attended with exhaustion and high fever, is the prevention or mitigation of delirium. With this object, he enjoins it to be given frequently, and in such quantities as experience has shown can be most easily assimilated, a good average dose being from two drachms to half an ounce, diluted with water, every hour, or even every half hour. He also insists upon its early exhibition under such circumstances, and of its action being aided by nutritious food and suitable anodynes, to allay pain, induce sleep, and tranquilize the affected structures.

Such, in few words, is Toddism. In what essential respects does it differ from Brunonianism? Not in a single one, so far as treatment is concerned. The whisky juts out equally prominently in both systems, although Dr. Todd doubtless administered it with more judgment than his ingenious but unfortunate prototype.

I come now to consider very briefly the two great questions proposed for discussion in this paper, namely: First, is there really, as has of late been so frequently alleged, a change in the type of disease? and secondly, is it a fact that people are no longer able to bear the same depletory measures, when laboring under inflammation, as formerly?

In regard to the first of these questions, I unhesitatingly reply in the negative. I have certainly not seen anything, in a practice of upwards of thirty years, to warrant such an opinion. To me diseases appear as they did when I entered upon the study of medicine. I am speaking now more especially of inflammatory affections, whether idiopathic or traumatic, as they ordinarily manifest themselves to us in the daily routine of practice, and not of epidemics, which, as is well known, always wear a peculiar livery in consonance with the peculiar nature of their exciting causes. These diseases, comprising erysipelas, typhoid fever, scarlatina, measles, small-pox, cholera, yellow fever, pneumonia, and other kindred affections, are seldom precisely alike during any two successive visitations. They are subject to constant fluctuations, arising without any assignable cause, leading to remarkable variations in their mortality, and requiring essential modifications of treatment. But there is no change of type, properly so called, even here. Whoever will take the trouble to inquire into the history of epidemics, as they have prevailed in different ages and in different countries, cannot fail to be impressed with the truth of this remark. To refer only to one of these epidemics, scarlet fever, who does not know that it is at one time so violent as to decimate an entire community, or destroy entire families, while at another it is so slight as to pass almost harmlessly over an entire country?

But if we take the ordinary inflammatory diseases, idiopathic and traumatic, of the human race, those which are liable to assail all persons, of whatever age, rank, or occupation, the only changes to which they are liable are such as are dependent upon individual peculiarities, growing out of their mode of living; that is, the nature of their food and drink, their residence, occupation, and social relations, and the state of their health at the time of their seizure, more particularly the state of the blood, the secretions, and the digestive apparatus. All these circumstances modify disease, and consequently demand a corresponding change of treatment. But in point of fact the morbid action is essentially the same; it may vary in degree but not in character. Its type, its essence, is the same. The blood-vessels at the seat of the disease are in the same condition; the deposits are of the same nature; the vessels may be more distended or less distended, more brittle or less brittle; the serum may be more abundant in one case than in another; the lymph may be plastic or aplastic; the pus may be laudable, sanguinolent, or fibrinous. These are mere individual differences, dependent upon accidental circumstances and not upon any changes in the type of disease. A furuncle, a carbuncle, an erysipelas, a whitlow, a conjunctivitis, exhibit the same characters, both outwardly and inwardly, now that they did ages ago; and if this be true of the external inflammations, may it not be justly inferred that it is true also of the internal, as pneumonia, pleurisy, hepatitis, cystitis, gout, and rheumatism? I can myself discover no differences between the inflammations which supervene upon wounds, sprains, contusions, fractures, and dislocations of the present day and those which were wont to meet my eye from these causes in my early professional life, save, of course, what are due to individual peculiarities. Their type is essentially the same now as then. Recovery and death are governed in both forms of these inflammations—the idiopathic and the traumatic—precisely by the same laws. The same causes which were in operation in producing ordinary inflammation thirty years ago, are in operation now, and the results must therefore be similar. Our habits and modes of life, our food and drink, our emotions and passions, are the same to-day as yesterday. The same sun and moon and stars afford us light and heat; we walk upon the same earth; we breathe the same air. Nature has not been upheaved by any unusual convulsions; the laws of God move on in their accustomed order.

There is no evidence, certainly not on this side of the Atlantic, of any serious physical degeneracy of the race in any walk or pursuit of life. The gardener, the plowman, and the dairymaid are as hale and robust as they have ever been. The blacksmith's brawny arm makes as much noise with the hammer upon the anvil as in the days of his ancestors. The carpenter, the mason, and the drayman have shown no diminution of manly vigor. The soldier wields the sword and musket with as much ease as he ever has; and the mariner is as justly entitled now to the appellation of "hardy" as he was at the battle of Aboukir, Trafalgar, or Lake Erie. Man's weight and stature, muscular development, and the quantity of his blood, have undergone no perceptible change within the last thirty years.

From all these facts, we infer that ordinary diseases are incapable of undergoing any cyclical changes of type, and that what have been regarded as such have had their origin in the mind of the physician and not in the body of the patient. In short, that all such opinions are imaginary and not real.

Brunonianism was essentially based upon the assumed inability of the people to bear depletory measures when laboring under inflammation; and yet it must strike every one as a most singular coincidence that, while the practitioners in many parts of Europe were pouring stimulants by the wholesale into the stomachs of their patients, Rush of Philadelphia was indulging in the most copious abstractions of blood, even during the epidemic yellow fever of 1793 and 1797, and busily engaged in laying the foundation of the bleeding school, whose influence was destined to last the better part of the third of a century. Surely no one will contend that this difference in the practice of the two countries at that period depended upon any material difference in the constitution of the people, or change in the type of disease.

The great error of the Todd school is the assumption that all diseases are due to a toxic condition of the blood. That some poison or other does exist in the system in certain affections, as erysipelas, scarlatina, pyæmia, and typhoid fever, is highly probable; at all events, such an opinion is now very common among physicians; but it is surprising that a man of the mental acumen of Dr. Todd should have made a general application of such a doctrine, and thence drawn the conclusion that all diseases require a stimulating plan of treatment in every stage of their progress.

Entertaining these convictions, I need hardly say that the idea that all inflammatory diseases should be treated with stimulants, has not only no foundation in truth, but is at direct variance with the best interests of the sick, and the reputation of the physician, considered as an enlightened, honest, and conscientious man. It can be clearly shown that such an exclusive system of medication is based either upon superficial observation and hasty generalization, or, what is worse and more reprehensible, upon a desire, on the part of those who have given currency to it, to become the founders of a new sect of medicine, with a view of gratifying selfish and ambitious purposes. It is difficult to conceive how any physician, enlightened by clinical experience, could bring himself by any process of reasoning or of logic to such a conclusion. If the opinion were a mere abstraction, it might be regarded as an ingenious quibble, calculated to excite our curiosity, perhaps our risibility; but it is very different when it is presented for our adoption at the bedside. It then assumes a more imposing attitude, and exhibits itself in all its naked deformity, as a monster ready to ingulf whoever is credulous enough to put his trust in the skill of the physician.

The only rational mode of treating disease is to individualize every case and to permit it to make its own laws, these laws having reference to the nature of the exciting cause, the peculiar structure involved, the period that has elapsed since the commencement of the attack, and, lastly, though not least, the state of the system immediately preceding the outbreak of the malady, as well as at the time of the physician's first visit. It is only by such considerations as these that the practitioner can hope to do full justice to his patient, and to preserve the honor and dignity of Hippocratic medicine. He must not view disease through the murky eye of prejudice, or approach the bedside with his mind filled with preconceived notions of debility or plethora; but he should so examine the case as to be able to give a just and satisfactory reason for the faith that is in him. If the stimulant school see nothing but reduced action or depressed vital power, it proves pretty conclusively one of three things, namely: either that they are incapable of making accurate observation, that they are blinded by prejudice, or that they have to deal with a class of patients whose habits and conditions are such as to forbid the employment of any other method of cure.

But we can certainly not suppose that the great advocates of the stimulant mode of treatment are not accurate observers; they are men of too much ability, mental discipline, and sober judgment to lend coloring to such an idea. Shall we accuse them of prejudice? All men are more or less under the influence of this passion, and it may, therefore, be assumed that the wisest and best of the advocates of this doctrine are not altogether exempt from it; nevertheless, no one could justly ascribe to them such a sin. What, then, is the source of this practice? Discarding these two conjectures as untenable, are we to conclude that these pathologists have no other than broken-down, worn-out, or decrepit patients, the residents of crowded cities, and the inmates of foul, ill-ventilated hospitals? Here, again, our proof is at fault; for while it may be supposed that both Todd and Bennett saw mostly persons of this description, it would be absurd to imagine that they had not also sometimes met with a stout, robust one, capable of bearing almost any amount of depletion and starvation. Has not Dr. Bennett himself afforded a complete refutation of this supposition in his own case of pneumonia, for the relief of which he was so copiously bled by one of his colleagues? Is it the stage of the disease that has produced this change of sentiment? Here, again, there is nothing positive, although it may reasonably be inferred that these practitioners are not always called in after the disease has committed such ravages as to admit of relief only by supporting measures.

It is well known that fashion has its influence in our art as in everything else. An ingenious tailor or mantua-maker often revolutionizes, by a mere stroke of the scissors or the addition of a trifling embellishment, the costume of an entire nation. It has already been seen how a dissolute doctor, who for many years hardly ever drew a sober breath, and who probably never had a hundred patients in his life, became the founder of a new sect in medicine, which maintained its sway for nearly a quarter of a century, its sole basis being the relief which he experienced in his own person from alcoholic potations and laudanum, whenever he suffered from an attack of gout, to which, for many years, he was a victim. Unity of disease and the lancet were the great weapons in the hands of our illustrious countryman, Rush. Purgatives were all the fashion in the days of Hamilton. Dyspepsia was the absorbing complaint with Abernethy and Phillip. In the time of Faithorne and Johnson every body had disease of the liver; and it is within the recollection of the elder members of the profession what an influence the doctrine of spinal irritation exercised upon the minds of practitioners, both in the Old World and the New, in the moulding of their views of pathology and practice. Broussaisism strutted for awhile triumphantly upon the stage on the basis of gastro-enteritis, leeches, ptisans, and gum-water. Cookeism rejoiced in an obstructed portal circulation, in vitiated bile, and in the R. A. C. pill, as it was called, a compound of rhubarb, aloes, and calomel. With some everything is a change in the type of disease; and both with them and Todd and John Brown, the only safety for the patients is in alcohol and other kindred remedies. Bleed the patient and he dies; purge him actively, and if he is not killed outright, his recovery will be greatly retarded, and his constitution, perhaps, irreparably damaged. Just now, the cellular pathology of Virchow is coming into notice with all the authority and prestige of an eminent foreign teacher, and it is not difficult to foresee to what extent it is destined to enslave the medical mind of this and other countries.

Although most medical men, as well as the community at large, view blood-letting at the present day with a kind of holy horror, the remedy has been employed, millions upon millions of times, with the greatest benefit in various inflammatory affections, from Hippocrates down to the present hour. That it has been abused is unquestionable. We can hardly reconcile with our notions of propriety the loss of two, much less of three hundred, ounces of blood in the course of a few days for the cure of an idiopathic or traumatic inflammation; and yet cases of this kind of a well-authenticated character are easily found in the annals of medicine and surgery. Dr. Francis, of New York, distinguished alike as a skillful physician and an accomplished scholar, is a living monument of the efficacy of this heroic treatment. In a violent attack of croup he lost, in a few days, nearly three hundred ounces of blood. Physick, in an attack of yellow fever, in 1797, was bled to the amount of one hundred and seventy-six ounces; and the practice to which he himself submitted he did not hesitate to apply to his patients in all cases attended with inordinate vascular excitement. He rarely extracted a cataract without drawing blood from the arm at least once a day, or every other day, until all danger of undue inflammation was at an end. His success in this operation was proverbial.

No practitioner at the present day would dare to bleed to anything like such an extent; and yet there could doubtless be found in every community men who would bear up well under it. But bleeding is no longer the fashion; the lancet is growing rusty in its case, and the cutler has almost ceased to manufacture it. Even the occupation of the leecher is almost gone. When I was a student in this city, it was very common in attacks of acute pleuritis to bleed a patient three or four times, and sometimes, if the suffering was very intense, perhaps even that often a day, the same orifice being repeatedly reopened for the purpose.

If we inquire into the causes of these revolutions in medical opinion, we shall find them, not in a change in the type of disease, or in an actual change in the powers of the system to withstand the effects of inflammation, but in a greatly enlarged knowledge of disease and of the virtues and application of remedies. The use of quinine and morphia is incomparably better understood now than it was thirty years ago; and as to aconite and veratrum viride, so highly esteemed at the present day in the treatment of inflammation, their antiphlogistic properties were entirely unknown until within a very recent period. Quinine has become the great remedy in all malarial maladies; opium is a powerful adjuvant in allaying pain and insuring rest both to part and system; and aconite and veratrum viride exercise so astonishing an influence in controlling arterial action as to render them special favorites with every enlightened practitioner. They have, in fact, become to be regarded as admirable substitutes for the lancet, tartar emetic, calomel, and active purgatives. Thus it is easy to perceive why blood-letting has fallen into desuetude; unmerited certainly in many cases, for, say and do what we please, there is no doubt that an early spoliative bleeding and the judicious use of the more heroic articles of the materia medica, formerly so much in vogue, will often do more in saving structure and function than any other of the popular remedies of the present day.

Practitioners are too prone to run into extremes, and to be led away by every specious theory wafted across the mind by men of genius, occupying high places, and speaking with the force of authority. Losing sight of common sense, and forgetting the teachings of experience, they are ever ready to grasp the shadow and forsake the substance. Hence the discordant views which so constantly agitate our profession, perplexing the public mind, and impairing our influence as enlightened and scientific physicians. Hence the strong hold which homœopathy, hydropathy, Thompsonianism, and other kindred delusions have taken upon the people. We constantly talk of the sins of our neighbors, but do not see the beam that is in our own eyes.

The past is pregnant with useful lessons. It teaches us, on the one hand, the futility, the utter folly and vanity, of all exclusive systems of medicine; and, on the other, the importance of being governed, in our views of pathology and practice, by common sense and chastened experience tempered by judgment, which holds fast to whatever is good and useful in our art and discards whatever is hurtful. Its motto is enlightened, unprejudiced conservatism, ever ready to enlarge its boundaries by cautious observation and induction, and resolved to avoid exclusiveism in whatever guise it may present itself.

Standing upon this elevated and philosophical platform, rational medicine avails itself of whatever is useful in mitigating and curing disease; not disdaining, on the one hand, the employment of the lancet, tartar emetic, calomel, aconite, veratrum viride, and other depressants, when it has to deal with violent inflammatory excitement attended with a sthenic state of the system; or, on the other, a recourse to stimulants, as brandy, wine, quinine, opium, and nutritious food, when the system is worn out by disease, or when the morbid action is attended with exhaustion of the vital powers.

As the practitioner grows older and wiser, an enlightened skepticism commonly takes the place of his boasted infallibility; he distrusts many of the remedies in which he was wont implicitly to confide in early life; his calmer judgment readily detects the fallacious glare of the systems of the schools, and he adopts, with doubt and hesitation, the hasty generalizations of enthusiastic and misguided innovators. Formerly he had ten remedies for every disease; now he has hardly a single one for any. Conscious of the imperfections of his art, he administers medicine with a sparing hand, carefully eschewing everything that savors of polypharmacy and heroic measures; and cherishes a warmer respect for the resources of Nature, satisfied that, while he may often be her assistant, he can never be her master. The usurpations of youth have yielded to the dictates of sound philosophy; and, although he may not be wholly free from the temptations of novelty, his mind is too strongly fortified by the lessons of experience to be easily led astray by any system, however facile or specious. The tyrant, fashion, has no longer any influence over him; he looks warily at every new doctrine, and calmly and dispassionately inquires into its merits before he permits himself to adopt it. He knows that an adherence to principles, based upon a professed study of pathology and therapeutics, constitutes the crowning glory of the physician.

The danger, then, in regard to this so-called new doctrine, is not to the old practitioner, grown wise and wary by experience, but to the young and incautious, fresh from the lecture-room, fond of novelty, and easily captivated by those "upstarts" in the profession, to whom the world is too apt, in its thoughtlessness, to accord the title of genius, however little it may be deserved. Such men seldom have so well balanced a mind or so calm and philosophical a temper as to render them fit leaders in matters of science, especially in one of such a momentous nature as that of medicine. There is usually some great defect in their mental organization, an absence of some important link, which sooner or later becomes apparent even to their most blind adherents, and which is sure ultimately to produce their downfall. They may, indeed, for a time, meteor-like, dazzle the horizon of the profession, but their reign is generally short-lived; and so it will be with Toddism, or the exfodiated stimulant treatment, so speciously paraded by its gifted author for our sanction and adoption.

The school of John Brown, which at one time counted its followers by hundreds, if not thousands, survived hardly a quarter of a century. Broussaissism had a still shorter career. Cookeism also perished in a few years. In a word, all extremeism, all exclusiveism, of whatever character, is necessarily destined to a brief existence.

Young Physic, warned by the sad experience of the past, should calmly ponder and cautiously try, before it decides to accept, in its full extent, so pernicious a doctrine. It has a duty, a most solemn and momentous duty, to perform; and its great mission to humanity will be a failure and a mockery, if it does not boldly set its face against all exclusive systems in medicine. "Soldiers!" said Napoleon, addressing his troops in Egypt, "Soldiers! from the summit of those Pyramids forty ages are looking down upon you!" "England," exclaimed Nelson, speaking to his brave mariners by signals on the eve of one of the most bloody naval engagements the world has ever witnessed, "England expects every man to do his duty." Young Physic! Hippocrates, the father of medicine, is looking down upon you through the long vista of nearly twenty-five centuries, and is beckoning to you to do your duty to your profession and to your fellow-beings.

Finally, let those who are so ready to put their trust in new systems of medicine, remember the lines of Ovid:—

> "*Nec lex est æquior ulla;*
> *Quam necis artificem arte perire sua.*"

